# Commentary: The Risky Closed Economy: A Holistic, Longitudinal Approach to Studying Fear and Anxiety in Rodents

**DOI:** 10.3389/fnbeh.2021.664941

**Published:** 2021-03-25

**Authors:** Neil Scheidwasser, Melissa Faggella, Elizaveta Kozlova, Carmen Sandi

**Affiliations:** Brain Mind Institute, École Polytechnique Fédérale de Lausanne, Lausanne, Switzerland

**Keywords:** fear, anxiety, decision-making, methods, ethology

Animals must constantly adapt their behavior as they navigate through their environment. For instance, fear and anxiety are essential to survival because they elicit immediate responses to harm. Whereas, fear denotes a transient state that arises in response to looming, tangible threats, anxiety-like reactions generally emerge in anticipation of a potential, albeit not concrete danger (Davis et al., 2010). Nowadays, research on emotion regulation and processing is heavily focused on fear and anxiety. Both have been found to be dysfunctional in a variety of human mental conditions, e.g., depression and post-traumatic stress disorders (American Psychiatric Association, 2013). These defensive behaviors are also highly conserved across mammals (Tovote et al., 2015). As a result, many experimental tasks have been developed to delve deeper into the neural basis of fear learning. Through a comprehensive review of the most popular paradigms for rodents, recent work by Schuessler et al. (2020) discusses the potential of the “Risky Closed Economy” (RCE) as a more realistic and holistic paradigm to study fear and anxiety. While previously employed in rats (Helmstetter and Fanselow, 1993; Kim et al., 2014; Pellman et al., 2015), the authors also explore a possible adaptation of the RCE for mice.

In contrast to standard rodent experimental paradigms [e.g., Pavlovian fear conditioning (Pavlov, 1927), inhibitory avoidance (Wilensky et al., 2000)], closed economy setups do not allow any nutritional supply outside of the behavioral chamber. In other words, animals must continually acquire their food and water during operant sessions, which results in enhanced involvement of the individuals during the tasks (Kearns, 2019). Schuessler et al.'s RCE's design includes a separation between the animal's nest and a risky “foraging zone,” where potential threats (e.g., predators) are modeled by random footshocks. All in all, these features aim at better approximating natural foraging conditions for rodents (Lima and Valone, 1986).

Another advantageous consequence of the RCE setup is that experiments can be implemented over significantly larger time scales. While fear conditioning experiments usually span only from minutes to a few hours (Pellow et al., 1985; Maren, 2008), Schuessler et al. (2020) were able to monitor mice behavior 23 h/day for 7 weeks. The longer autonomy given to animals helps minimize observer-expectancy effects, as experimenters only intervened for daily health checks. Moreover, studying behavior quasi-continuously during extended periods enables a better overview of how an animal's fear and anxiety evolve over time.

Although the RCE has only been previously applied to rats (Helmstetter and Fanselow, 1993; Kim et al., 2014; Pellman et al., 2015), Schuessler et al. (2020) showed that it can also be adapted to mice. That development will likely open up more research opportunities, in particular making optogenetics experiments easier and drug testing more efficient. However, the success of the current implementation is somewhat mixed. While some of the results from previous rat experiments were successfully replicated, others were not. For instance, introducing unsignaled footshocks in male mice decreased their foraging time, but did not influence the meal size, whereas rats (male or female) showed a change in both parameters (Helmstetter and Fanselow, 1993; Pellman et al., 2017). As a matter of fact, the authors rightfully acknowledge that the footshock regime was likely to be maladapted to male mice. To that end, future work using the RCE should be performed to find more suitable threat paradigms for mice. In addition to the footshock regime, the RCE's viability in mice could also be extended by including signaled footshock paradigms (Pellman et al., 2015) and assessing sex differences in behavioral response to the paradigm. Noting the sexual dimorphisms exhibited by rats in similar closed economy setups (Pellman et al., 2017), or by mice in contextual fear conditioning (Wiltgen et al., 2001; Colon and Poulos, 2020), similar gender disparities seem likely in mice within the RCE.

More generally, several approaches could be implemented to further improve the RCE's ethological relevance for all rodents. For instance, a study of stress hormone dynamics would enhance our understanding of the results yielded by this paradigm. This could be done non-invasively by measuring corticosterone in fecal samples (Touma et al., 2004) to avoid disrupting the RCE environment. Next, using predator-derived odors (e.g., from feces or urine) instead of footshocks would exploit the fact that mice are macrosmatic animals (Gire et al., 2016), and could thus represent a more accurate approximation of potential harm. In addition, as mentioned by the authors, it would also seem natural to incorporate some degree of social interaction, given the social nature of rodents (Tamashiro et al., 2005). Group interactions have already been successfully introduced into similar naturalistic paradigms in other domains (Karamihalev et al., 2019; Kiryk et al., 2020). What's more, recent advances in deep learning make analyses of complex data emerging from these settings easier to handle than ever before. Animal tracking has reached very high accuracy, especially in static environments like laboratory cages (Forys et al., 2018; Mathis et al., 2018). Moreover, automated pipelines such as the Mouse Action Recognition System (MARS) (Segalin et al., 2020) can classify specific types of social interaction in rats with human-level accuracy. Such frameworks could certainly enrich the analysis of RCE experiments with complex behavioral data such as emotional responses (e.g., anxiety-like and depression-like behaviors). That in combination with stress hormone levels would allow understanding whether the RCE paradigm is able to induce and/or assess emotional dysfunctions. Such an approach would facilitate the use of this environment on studies that explore the impact of drugs, or other treatments, in interaction with the living environment (see, e.g., Alboni et al., 2017). Future research will also need to adapt state-of-the-art neurophysiological techniques like optogenetics, fiber photometry; mini-endoscopes for such longitudinal paradigms to shed more light on the neural mechanisms of fear, anxiety, and other relevant behaviors. All in all, although more time-consuming than most fear conditioning paradigms, the RCE enables multi-variable behavioral analysis in a more naturalistic environment. The variety of possible combinations with other methods and technologies makes this paradigm a promising alternative to study fear and anxiety in rodents.

## Author Contributions

NS wrote the first draft. NS, MF, EK, and CS edited and contributed to the writing of the final draft. All authors contributed to the article and approved the submitted version.

## Conflict of Interest

The authors declare that the research was conducted in the absence of any commercial or financial relationships that could be construed as a potential conflict of interest.
